# A new device for the removal of cochlear schwannoma: A temporal bone study

**DOI:** 10.3389/fsurg.2023.1077407

**Published:** 2023-02-01

**Authors:** Holger Sudhoff, Conrad Riemann, Rayoung Kim, Lars Uwe Scholtz, Christoph J. Pfeiffer, Peter Goon, Ingo Todt

**Affiliations:** Department of Otolaryngology, Head and Neck Surgery Medical School OWL, Bielefeld University, Campus Mitte, Klinikum Bielefeld, Bielefeld, Germany

**Keywords:** cochlear implant, intracochlear intralabyrinthine schwannoma, tissue removal device, temporal bone, hearing implant

## Abstract

**Background:**

Intralabyrinthine schwannoma (ILS) is a rare, mostly unilateral disease that causes deafness. Different intralabyrinthine sites of ILS can occur and can be removed by different surgical approaches. Cochlear ILSs are frequently partially hidden by the modiolus and therefore difficult to extirpate. Surgical techniques can be traumatic, offer limited surgical control during removal, and are time-consuming. The aim of this present study was to demonstrate the performance and handling of a newly developed device for the removal of cochlear intralabyrinthine schwannoma in the temporal bone.

**Methods:**

In a temporal bone study with a prepared posterior tympanotomy, an enlarged round window approach, and additional second turn access, a stiffened device with silicone rings was inserted and extracted gradually from the second turn access until the rings were visible in the second turn access.

**Results:**

Insertion and extraction of the second cochlear access were easily performed. Pulling and pushing the silicone rings through the modiolus and hidden parts of the basal turn was possible and worked like a pipe cleaner.

**Conclusion:**

This newly developed tissue removal device in combination with the proposed surgical handling offers a new and less traumatic way to remove cochlear ILS.

## Introduction

Intra-labyrinthine schwannomas (ILSs) are rare tumors of the lateral skull base and can cause unilateral deafness and can occur in neurofibromatosis type II (NFII) (1, 2). These tumors have been classified by Kennedy et al. (3) according to their location. The authors differ between cochlear, vestibulocochlear, vestibular, transmodiolar, and other subtypes of ILSs (3, 4). After successful removal, cochlear implantation is the treatment of choice for rehabilitating binaural hearing (5, 6). Tumors expanding to the IAC need a different treatment concept balancing out surgical removal, wait and scan, and radiation surgery.

The localization of the tumor determines the surgical approach. Different techniques have been described to perform an individualized tumor localization-dependent surgical approach. ILSs localized or partially positioned in the vestibulum or the semicircular canals are accessed through labyrinthectomy (7). If an ILS can be overseen in the basal turn, it is removed by an enlarged cochleostomy approach. ILSs in the first turn and hidden by the modiolus can be accessed by additional cochleostomy, laterally above the oval window.

The aim of the present study was to demonstrate the handling and performance of a newly developed device for the removal of cochlear intralabyrinthine schwannomas in the temporal bone.

## Materials and methods

**Figure 1:**
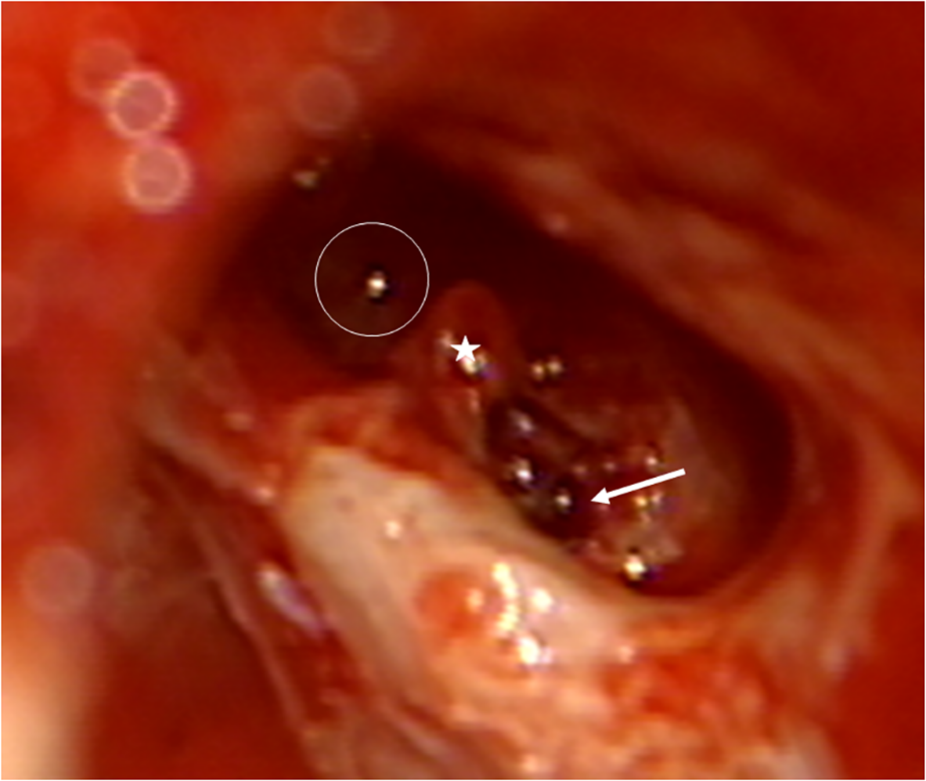
Access to the second turn in the right temporal bone. Arrow: basal turn cochleostomy, star: stapes, circle: second turn access.

**Figure 2:**
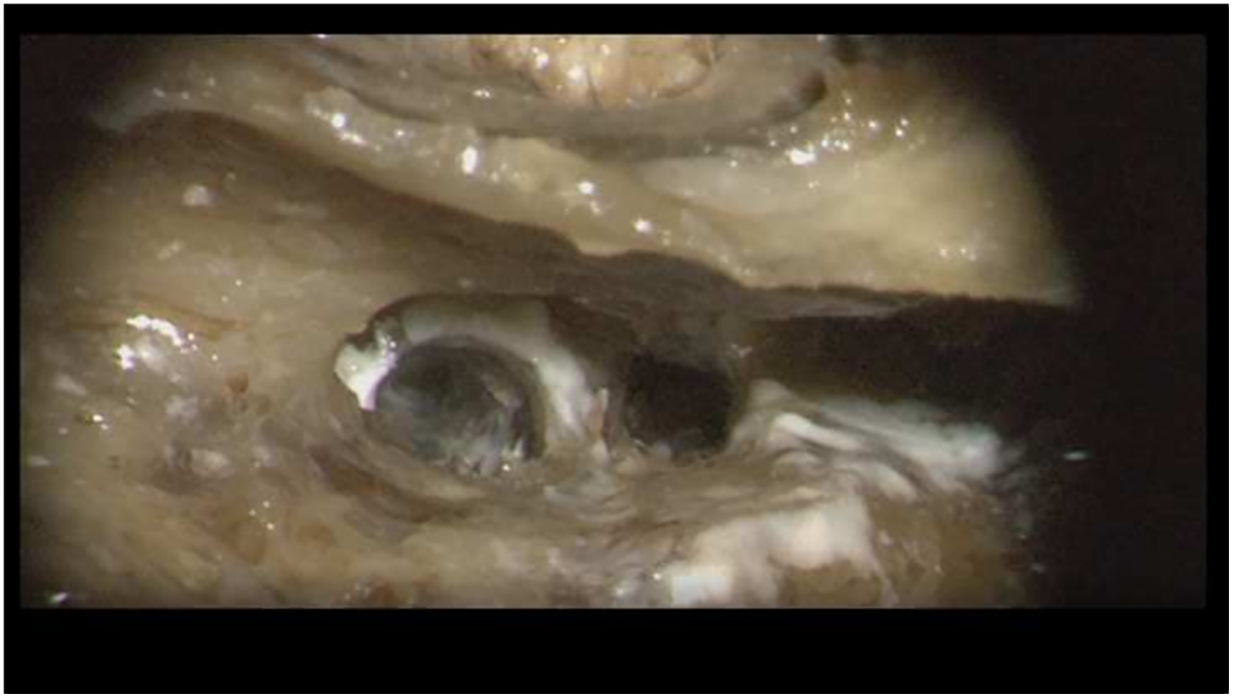
Posterior tympanotomy with cochleostomy and second turn access in the left temporal bone.

In this temporal bone study, a human temporal bone was prepared as performed in the regular surgical manner to remove cochlear ILS through posterior tympanotomy after the removal of the incus. Enlarged cochleostomy was burred, and additional access to the second turn of the cochlea was performed through posterior tympanotomy about 1.5 mm caudally from the facial nerve and 1 mm above the oval window, as described by others (Figure 1 and Figure 2) (11).

**Figure 3:**
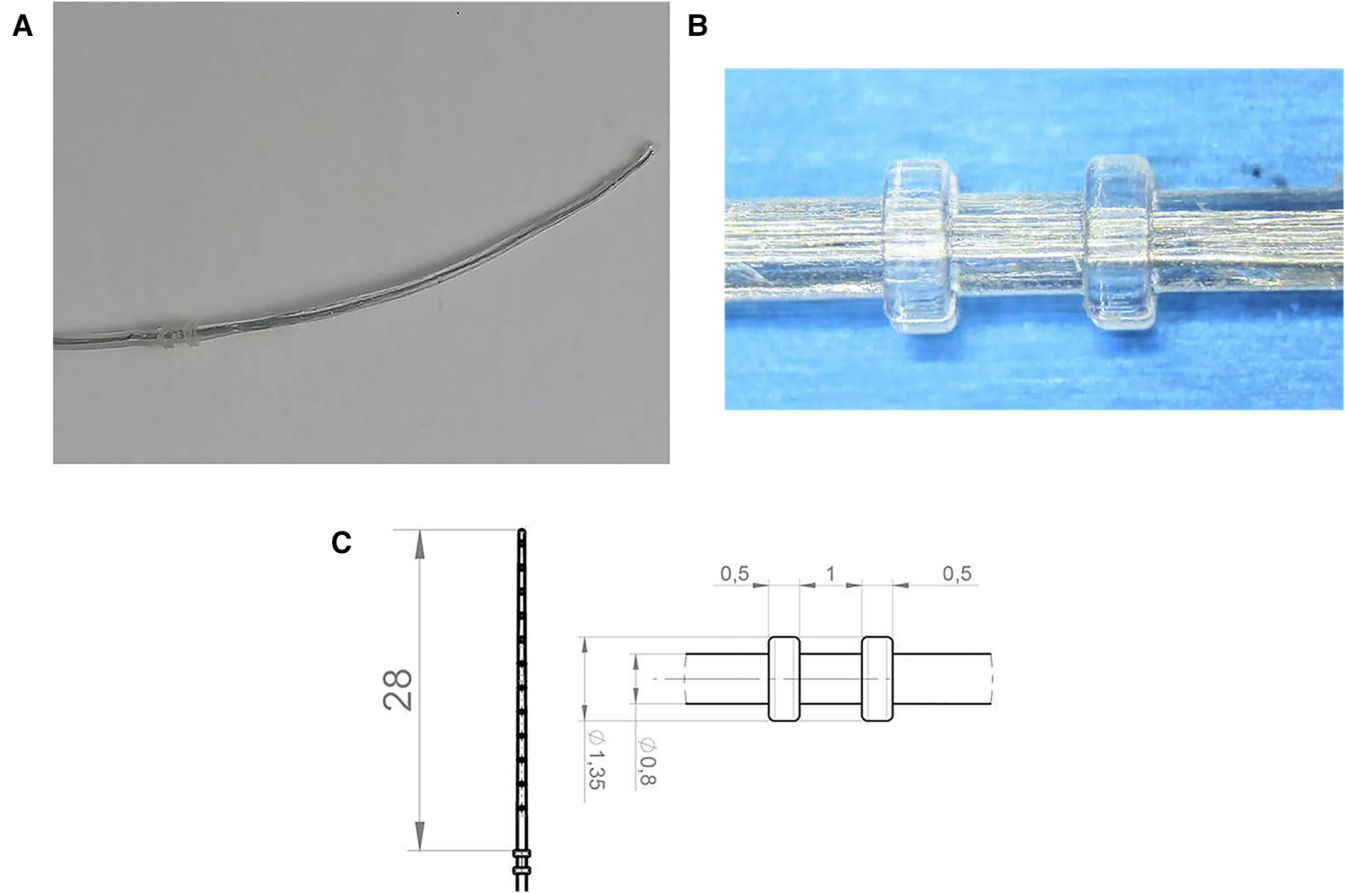
(**A**) Tissue removal device (TRD). (**B**) Two silicone rings. (**C**) Technical drawing including dimensions of the device in mm.

The new silicone-based tissue removal device (TRD) resembled the outer shape of a CI electrode array and was developed by Roland Hessler, Stefan Raggl (MED-EL Company), and the last author. It was produced as a custom-made electrode. It was mechanically reinforced and included two additional silicone rings with a diameter of 1.35 mm placed at 28 mm from the tip of the device. These rings were 1 mm apart and 0.5 mm in width (Figure 3A–C).

## Results

**Figure 4:**
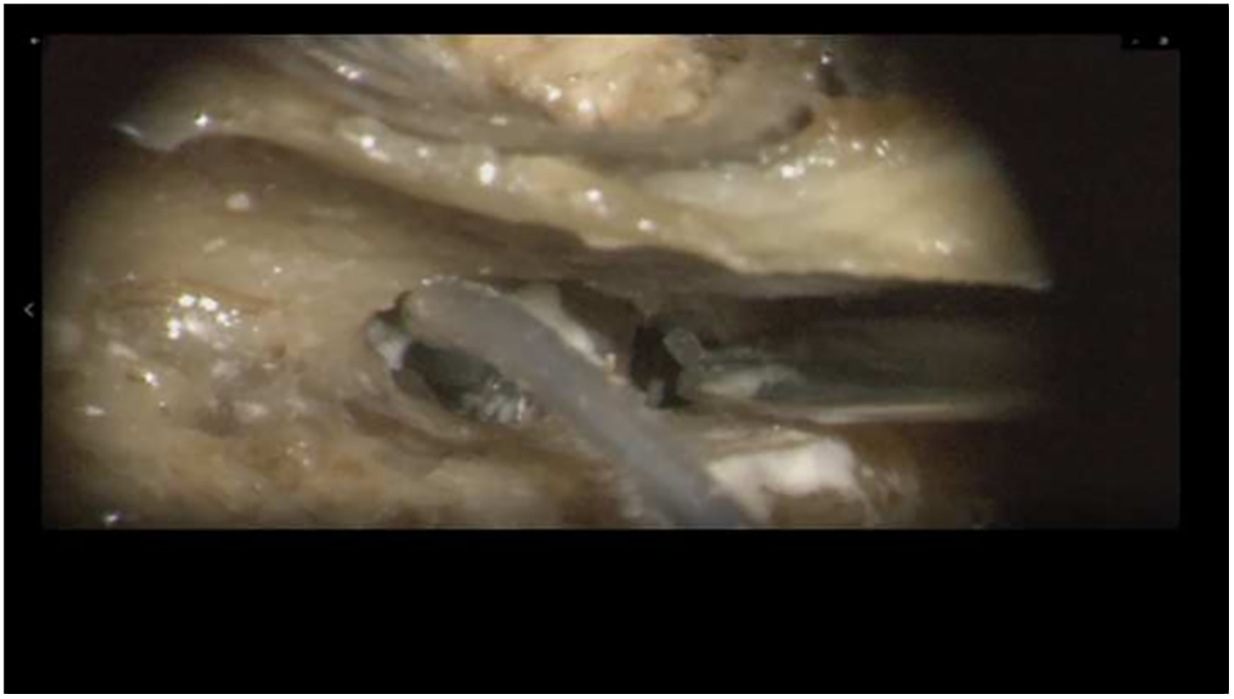
Pulling out the device tip.

**Figure 5:**
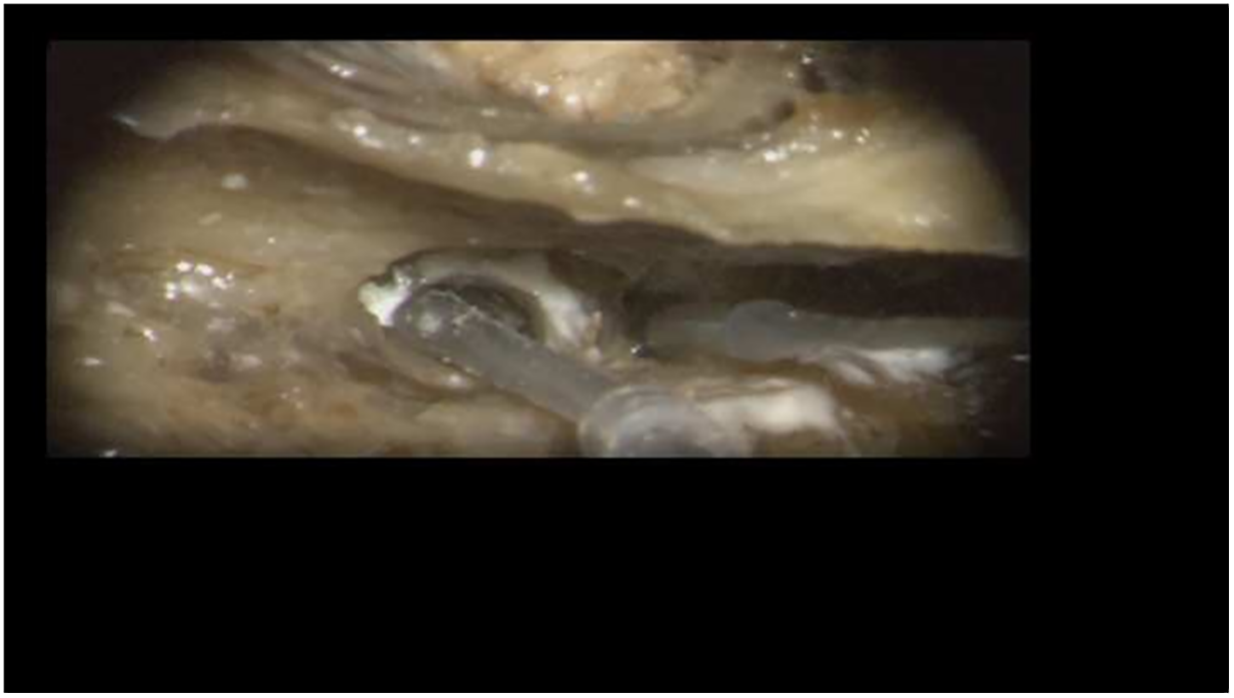
First silicone ring is visible in the cochleostomy.

**Figure 6:**
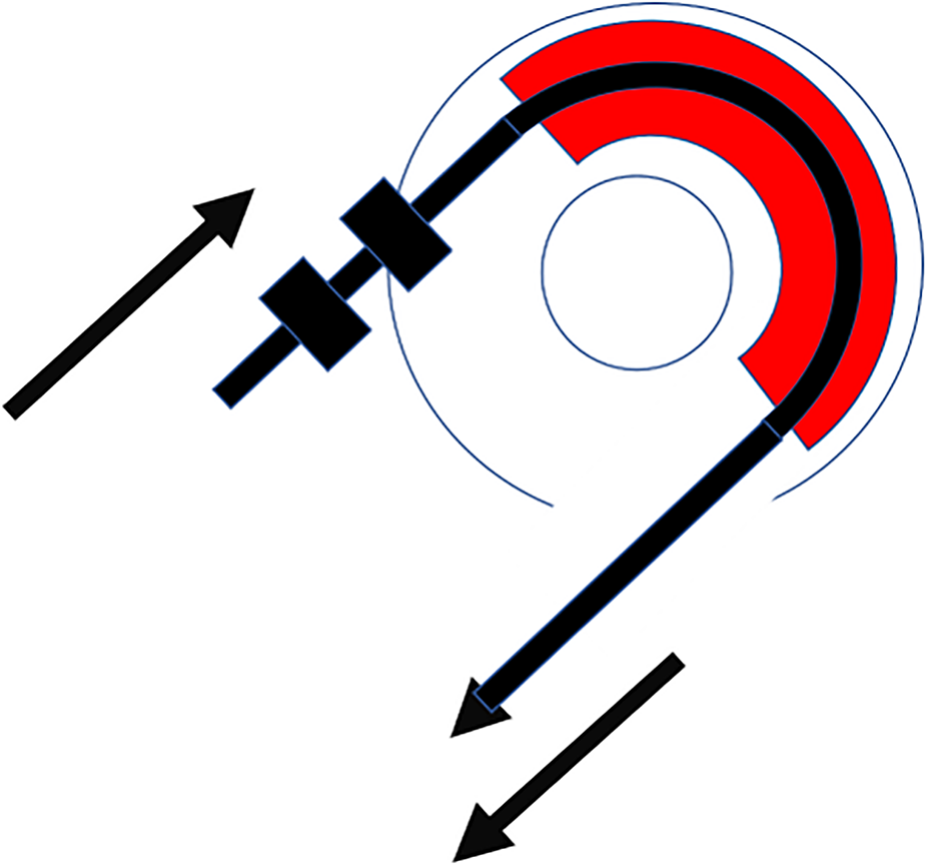
Schematic insertion of the device through the tumor (red).

**Figure 7:**
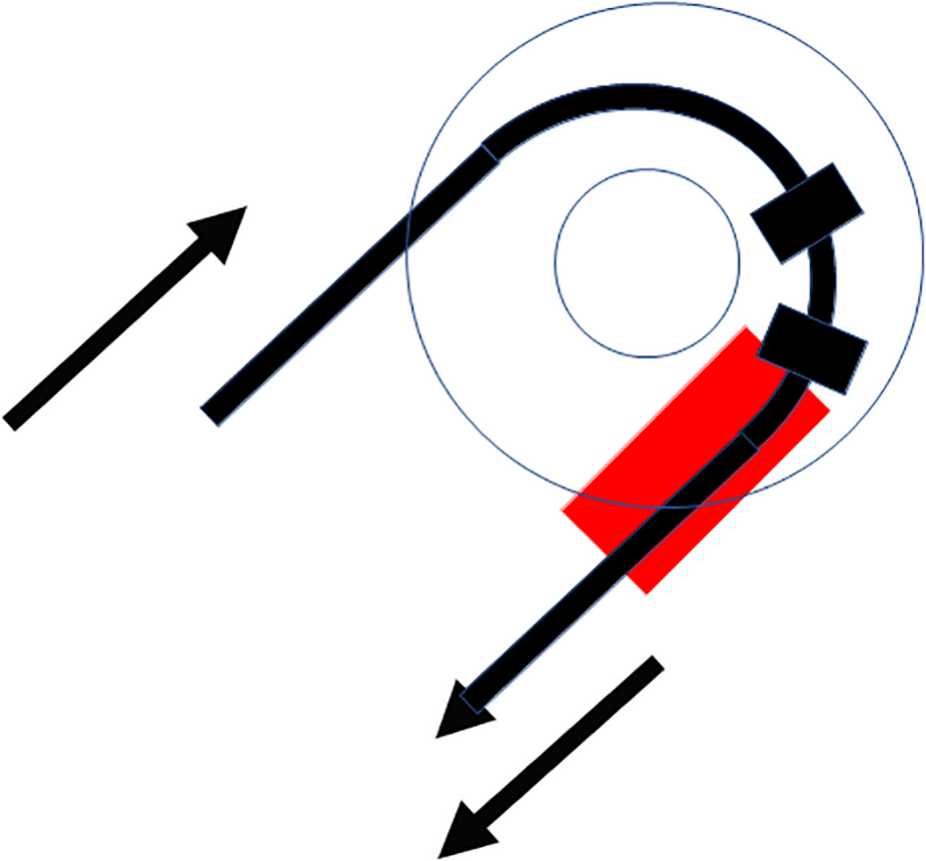
Schematic moving the tumor (red) out of the cochlea.

**Figure 8:**
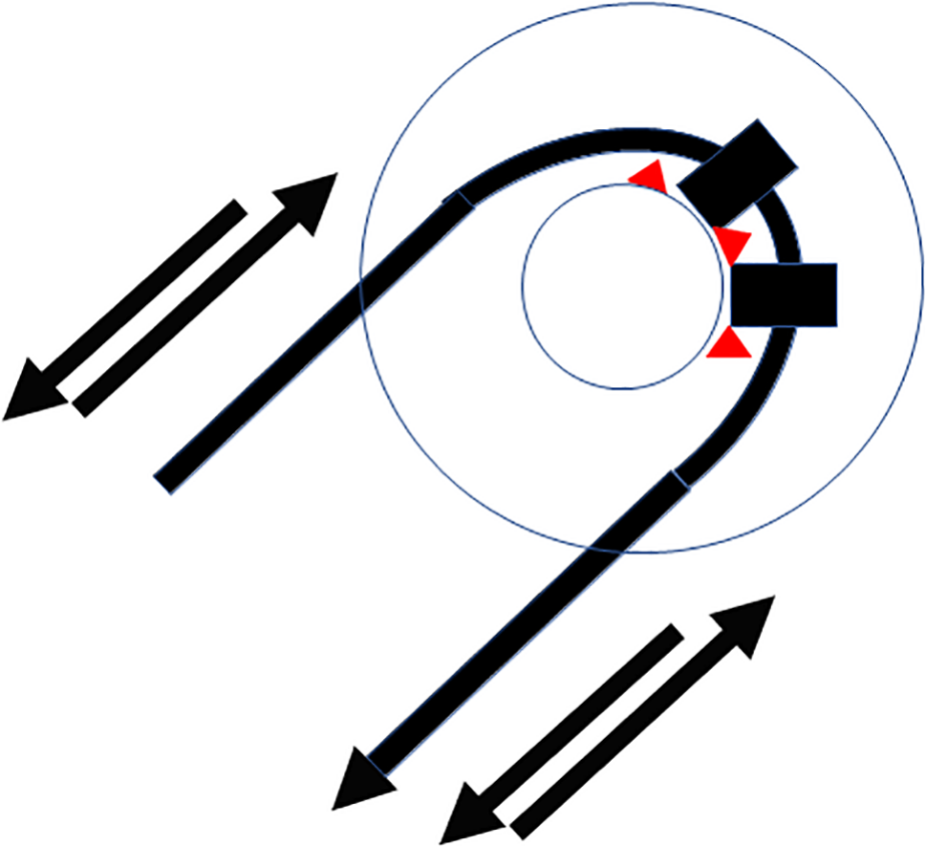
Schematic pipe cleaner handling of the device to remove residual tumor parts (red).

Insertion of the TRD was performed until the tip of the device was visible in the second access to the cochlea. Then, the tip of the TRD was caught or captured (Figure 4) and pulled out of the second cochleostomy until the first ring was visible (Figure 5). Now, the tumor-inserted electrode could be captured carefully at both ends (Figure 6). Afterward, the tumor could be removed completely or in parts (Figure 7) *via* a pulling and pushing motion inside the cochlear turn, similarly to a pipe cleaner (Figure 8), to remove residual parts.

## Discussion

Intralabyrinthine schwannomas are tumors of the lateral skull base that can occur spontaneously or as part of an NFII (1). With the increasing number of patients with unilateral deafness implanted with a cochlear implant and thereforea mandatory MRI in most countries, the occurrence of ILS has received much attention since more cases are found. Cases of patients with ILS have shown good hearing rehabilitation results with cochlear implants after tumor removal (10). In terms of the growing behavior of ILS, the literature is inconsistent. While some groups observed growth of ILS, others interpreted their data in the opposite direction (12–16). Therefore, there are groups that perform cochlear implantation without the removal of ILS (2, 17) and others that prefer to remove the tumor. Since in-growth of ILSs into the IAC fundus and transmodiolar ILSs are known and long-term growth is not excluded, the authors strongly recommend the removal of these tumors. Regarding the techniques, several methods have been described. Following surgical principles, ILS removal should be complete, easy to handle, atraumatic, and not time-consuming.

The completeness of removal of all techniques needs to be re-evaluated since quality control for these tumors has recently been made possible with specific considerations of behavior of implant magnets and MRI-generated artifacts (8, 18). Aschendorff described the usage of the so-called dummy electrodes (Contour Advance, Cochlear, Sydney, Australia), which followed, to some degree, the principle of the newly developed device. The stylet stiffened dummy electrode was inserted through enlarged cochleostomy, pushing the tumor to the second cochlear access, from where it was caught with forceps. The device shown in this study differs from these previously used dummy electrodes in terms of the outer silicone ring diameter (1.35 mm) and length of the electrode. Therefore, it can be assumed to be less traumatic to the intracochlear structures since it is adjusted to a thinner scalar diameter and can easily be pulled through the second access. This pulling through and catching with forceps can be assumed to allow a real pipe cleaning action by pulling and pushing the silicone rings inside the cochlea and removing residual tumor parts.

The described gel foam techniques used by Sudhoff et al. (8) are characterized by pushing of dry gel foam into the enlarged cochleostomy until the tumor is pushed out of the second access. This technique is compared to the present technique, with the new electrode being time-consuming and more traumatic.

Performing a cochleostomy or drilling out allows a visible control of the tumor region but is traumatic (9, 10). To which degree traumaticity is a relevant factor for assessing techniques is a matter of discussion. Plontke et al. showed, in most cases, regular audiological results (40%–90% after 12 months of 65 dB monosyllabic understanding) even after traumatic procedures to the cochlea (9). Another technique is cutting a dummy depth gauge at the basal part of the electrode and inserting the electrode into the basal part first to push out the tumor to the second turn. This technique is easy and can be rapidly performed but might miss modiolar parts of the tumor by the sidewall-directed character of the electrode. As a next step, *in vivo* observations are planned to compare surgical handling and MRI assessment with previously used techniques and their results.

## Conclusion

The tissue removal device in combination with the proposed surgical handling offers a new and less traumatic way to remove cochlear ILSs.

## Data Availability

The original contributions presented in the study are included in the article/Supplementary Material; further inquiries can be directed to the corresponding author.
