# Corrigendum: A new device for the removal of cochlear schwannoma: A temporal bone study

**DOI:** 10.3389/fsurg.2023.1195473

**Published:** 2023-04-28

**Authors:** Holger Sudhoff, Conrad Riemann, Rayoung Kim, Lars Uwe Scholtz, Christoph J. Pfeiffer, Peter Goon, Ingo Todt

**Affiliations:** Department of Otolaryngology, Head and Neck Surgery Medical School OWL, Bielefeld University, Campus Mitte, Klinikum Bielefeld, Germany

**Keywords:** cochlear implant, intracochlear intralabyrinthine schwannoma, tissue removal device, temporal bone, hearing implant

A corrigendum on A new device for the removal of cochlear schwannoma: A temporal bone study By Sudhoff S, Riemann C, Kim R, Scholtz LU, Pfeiffer CJ, Goon P and Todt I. (2023) Front. Surg. 10:1077407. doi: 10.3389/fsurg.2023.1077407

Incorrect version: Sudhoff Holger, Riemann Conrad, Kim Rayoung, Lars Uwe Scholtz, Christoph J. Pfeiffer, Goon Peter and Todt Ingo*

Correct version: Holger Sudhoff, Conrad Riemann, Rayoung Kim, Lars Uwe Scholtz, Christoph J. Pfeiffer, Peter Goon and Ingo Todt*

Incorrect version:

Citation: Holger S, Conrad R, Rayoung K, Scholtz LU, Pfeiffer CJ, Peter G and Ingo T (2023) A new device for the removal of cochlear schwannoma: A temporal bone study. Front. Surg. 10:1077407. doi: 10.3389/fsurg.2023.1077407

Correct version:

Citation: Sudhoff H, Riemann C, Kim R, Scholtz LU, Pfeiffer CJ, Goon P and Todt I (2023) A new device for the removal of cochlear schwannoma: A temporal bone study. Front. Surg. 10:1077407. doi: 10.3389/fsurg.2023.1077407

The authors apologize for this error and state that this does not change the scientific conclusions of the article in any way. The original article has been updated.

